# Popular Study of Anatomy and Physiology

**Published:** 1834

**Authors:** 


### Art. VII.—Popular Study of Anatomy and Physiology.

*A projected school and college book of Anatomy and Physiology, designed for both sexes.* By the Editor.

We hope to be pardoned for appropriating a few pages to the publication of a section of the contemplated popular treatise on Anatomy and Physiology, announced in our last number. The object in giving this specimen, is to recall the attention of our readers to that undertaking, and by offering them a portion of it, taken indiscriminately, to enable them to judge of its adaptation to the capacities of those for whom it is designed.

In this attempt to promote the popular study of the branches of science just mentioned, the Editor feels the great importance of securing the approbation of the profession, and he would again respectfully solicit of his readers such remarks on the enterprize, as they may suppose are calculated to be useful. In style and manner the section here introduced, may be considered as a sample of the whole. At what time it will be ready for the press cannot be stated, as it must depend chiefly upon the professional engagements of the Author.

### CHAPTER II.

*Section 2.*

### OF THE SPINE.

1. If a person passes his finger down the middle of his back, he feels a row of hard points. They are called spines, or spinous processes, and from them the back bone itself, of which they are a part, has received the name of spine, or spinal column.

2. This column is not a single piece, but consists of 24 separate bones united together. Each piece is called a *vertebra*, from *verto* to turn; and animals that have a spine of this kind, as man, quadrupeds, birds, reptiles, and fishes, are called vertebral or vertebrated animals; while insects, and worms, which have no spines, are called invertebral animals. This is the first great division of the animal kingdom, and shows the connexion between anatomy and natural history.

3. Every vertebra has a body, which is like a piece of cylinder or rod, sawed across, and consequently it has two flat surfaces, fitting it for union with the vertebra above and below it. Behind, it has a bony arch, the ends of which are attached to the body of the bone, and form by its aid an irregular ring; and the succession of these rings, throughout the whole length of the spine, forms a canal or tube for the spinal marrow, as may be seen by the annexed plate, where the rod represents the place of the canal.

4. From the centre of the arch of each vertebra, the spinous process arises, and on each side of it there are three others, one called the lateral or transverse, to assist in attaching the ribs to the spine; the other two named the oblique, and designed to connect the vertebræ with each other.

5. Between the vertebræ there is a considerable mass of cartilage or firm gristle, which is compressible and elastic like cork. It adheres to the surfaces of the bones and serves to connect them; but they are still more firmly knit together by ligaments or strings, which pass from one vertebra to another on every side of the column.

6. The vertebræ are divided into three classes—those of the loins, of the back, and of the neck—called *lumbar*, *dorsal*, and *cervical*. The whole taken together are about one-third of the length of the individual, and those of the back are equal to those of the neck and loins, while these again are nearly equal to each other. Thus, if the person is six feet high, his spine is two feet long, the dorsal portion being twelve inches, the cervical six, and the lumbar six, or a little more. Any considerable departure from these proportions is attended with a corresponding injury to the personal appearance of the individual, a fact which should be known to statuaries and historical painters, and shows the light which anatomy sheds upon the fine arts.

7. The lumbar vertebræ are only five in number, but from their great thickness they make a part of the column rather longer than the seven vertebræ of the neck. The lowest is the largest of the whole spine—the upper is nearly of the same size with the dorsal vertebra which rests upon it. These latter, twelve in number, are intermediate in size between the lumbar and the cervical, the upper one being the smallest of the whole. A separate description of the different vertebræ, from their general likeness to each other, is not necessary, except of the two upper joints of the neck; an account of which cannot, however, be given with clearness till the bones of the head have been described.

8. We have spoken of the spine as composed of 24 bones, but in reality it comprises a greater number, for the bone on which the lowest lumbar vertebra rests, is, strictly speaking, a part of the spine—the part which connects it with the hip or pelvis.

9. This bone is called the *sacrum*. It is nearly triangular, with the point of the angle downwards, the edges to the sides of the pelvis, and the two flat surfaces directed backwards and forwards. The upper side, corresponding with the base of a triangle, is firmly connected by ligaments with the last lumbar vertebra. From this joint the sacrum turns backwards, and then forwards to its lower end, so as to be convex behind and concave before. The spinal marrow descends into the sacrum, where it terminates in a number of nerves, which go out through holes to the lower extremities. It has the appearance of four or five vertebræ consolidated together, but destitute of the oblique and lateral processes with which the true vertebræ are furnished.

10. The sacrum has a point slightly moveable, an inch or more in length, somewhat resembling the beak of a bird, and called the *coccyx*. In the infant it is composed of four small distinct bones, which in the adult are united into one. The tail bones of animals is the coccyx extended.

11. When viewed in front or rear the spine is observed to be straight, that is, it neither bends to the right nor left, But when looked at in profile, or sideways, as in figure 3, it is seen to be curved. From the bottom of the skull it begins to descend nearly perpondicular, but very soon curves backward till it comes between the broadest part of the shoulder blades, when it gradually advances forward to the middle of the loins, after which it inclines a little backwards to its termination on the sacrum, which curves forward.

12. Thus the neck naturally projects forward from the shoulders, these are a little rounded, and the loins present a hollow. This is the curve line of beauty in the trunk of the body, and shows that a spine which is too straight, is as much at variance with a graceful figure, as one that, within certain limits, is too crooked.

13. If the spine is unnaturally long in proportion to its thickness, the person is weak, but supple in his back. On the other hand, large vertebræ indicate strength, a fact well known to those who deal in horses.

14. Thus, there are natural differences in the strength and flexibility of the spines of different persons; but much of the varety in this respect which we see in society, is referable to our different employments. The farmer in the country, and the porter in the city, who lift and carry heavy weights, have the bones of the spine closely knit together, and exhibit strength without much suppleness; but stage performers, circus riders, and rope dancers, who habitually throw themselves into a great variety of attitudes, are capable of bending their spines to a degree that often excites astonishment.

15. Among different races of animals this diversity is still greater. Thus, the spine of the horse, with the exception of its cervical portion, has but little motion among its different parts, but posseses great strength, and can carry heavy burthens; while that of the black snake of our woods, or the *boa constrictor* of South America, is so flexible, that the animal is capable of coiling many times round its prey. A difference in the manner in which the vertebræ are united, is the chief cause of these varieties.

16. The uses of the spine require that it should be made up, as we find it, of a number of pieces, separated by cartilage, and bound together with ligaments. These uses are, first, to support the head, the upper extremities, the ribs and the breast bone, with all the soft parts which cover them; secondly, to afford a fixed point for the origin and insertion of the muscles of the trunk of the body, and the upper bones of the extremities; thirdly, to protect the spinal marrow.

17. If the spine had been a solid piece of bone, the various motions of the trunk of the body, which are necessary in our daily employments, could not have been performed; if it had consisted of two or three joints only, the acute bending which would have been required at them, must have injured the spinal marrow by compressing it; and if it had been made of cartilage, or any other flexible substance, its strength would not have been sufficient to sustain the weight of the upper parts of the body.

18. The cartilage or gristle which lies between the vertebræ, is quite as necessary as the great number of those bones; for when we walk, or run, or jump down, or fall on our feet from a height, the cartilages between the vertebræ, in consequence of being elastic, are compressed and pushed out from between them, so that the jar or concussion of the brain and the whole body, is, to a considerable degree, prevented.

19. This important object is still further effected by the manner in which the oblique processes of the different vertebræ are applied to, or rest upon each other. The two lower oblique processes of the different vertebræ, are, in a manner, wedged in between the two upper oblique processes of the vertebra below, so as to admit of a slight motion upwards and downwards, when the individual jumps or falls on his feet.

20. The curvature of the spine contributes to the same important end, as any one may assure himself, by observing the difference in the jar communicated to his hand, by successively striking the end of a straight and a bent rod upon a hard body, taking care to place his hand

*upon* the upper end. When the individual has been for a long time on his feet, the weight of the upper parts of the body, so compresses the elastic cartilages as to make them thinner than when he first arose, and thus he is taller in the morning than the evening.

21. The spine is liable to become bent out of its proper figure, by a variety of causes. While it is still growing, if the child does not take sufficient exercise, the ligaments and muscles which connect the vertebræ, will be weak and relaxed; and a curvature to the right or left, or rather, in different parts of it, to both sides, may take place. Sitting daily for a long time in one position, whether erect or leaning forward, may produce distortion, and even sleeping constantly on one side may have the same effect. In all of these cases the vertebræ may grow into a different shape from what is natural, and the deformity be irremediable.

22. A more lamentable distortion, however, is the consequence of disease in the bodies of one or more vertebræ. A slow inflammation is followed by softening and decay, to this succeeds absorption, and the bodies disappear, leaving their processes, and the bony arch to which they are attached, uninjured. The bodies of the adjoining vertebræ, above and below, then come together, and adhere; the disease is cured, but an angle is formed, and the projection of the spinous processes constitute the hunchbacks which we every day see in the streets.

23. This is generally a disease of early life, and parents should know, that a recumbent or lying position, which takes off the weight of the upper part of the body from the inflamed vertebræ, is indispensable to the cure, and be ready to co-operate with the physician in his efforts to confine the child to a horizontal posture.

24. A still more serious injury of the spine, is the partial separation or dislocation of two of the vertebræ, generally called a broken back, the consequence of external violence .By this accident the spinal marrow is compressed, and all the parts which are supplied with nerves from below that point lose both feeling and motion. If this dislocation takes place in the upper part of the neck, above where the nerves of respiration or breathing go off from the spinal marrow, speedy death is the inevitable consequence—a fact which should be known to members of the bar, and all others who may be called to inquire into the causes of death from injuries which involve the spine.

25. As age advances the cartilages between the vertebræ are partially absorbed, and get thinner and harder. Their bodies approach each other more closely, and the individual becomes "stoop shouldered." The spine loses much of its suppleness and elasticity, its motions become limited, and jumping or falling on the lower limbs from a height, imparts a much more dangerous jar to the brain and spinal marrow than in childhood and youth.

26. Even this imperfect account of the structure and uses of the spine must present it as an object of admiration. Its strength and pliancy; the vast variety of motions which it favors; the points which it presents for the origin and insertion of muscles; the strong connexion of one vertebra with another, by means of the oblique processes, cartilages and ligaments; the adaptation of its curves to the movements of the head and limbs; its elasticity; and the perfect protection it affords to the spinal marrow which lies in its canal, and sends out nearly all the nerves of the body, most clearly disclose that it was planned and built up by an architect of infinite wisdom.

